# Lignin/poly(butylene succinate) composites with antioxidant and antibacterial properties for potential biomedical applications

**DOI:** 10.1016/j.ijbiomac.2019.12.146

**Published:** 2020-02-15

**Authors:** Juan Domínguez-Robles, Eneko Larrañeta, Mun Leon Fong, Niamh K. Martin, Nicola J. Irwin, Pere Mutjé, Quim Tarrés, Marc Delgado-Aguilar

**Affiliations:** a School of Pharmacy, Queen's University Belfast, 97 Lisburn Road, Belfast BT9 7BL, UK; b Group LEPAMAP, Department of Chemical Engineering, University of Girona, c/M. Aurèlia Campmany 61, 17071 Girona, Spain

**Keywords:** Lignin, Poly(butylene succinate), Biomaterials

## Abstract

Lignin (LIG) is a renewable biopolymer with well-known antimicrobial and antioxidant properties. In the present work LIG was combined with poly(butylene succinate) (PBS), a biocompatible/biodegradable polymer, to obtain composites with antimicrobial and antioxidant properties. Hot melt extrusion was used to prepare composites containing up to 15% (w/w) of LIG. Water contact angle measurements suggested that the incorporation of LIG did not alter the wettability of the material. The material density increased slightly when LIG was incorporated (<1%). Moreover, the melt flow index test showed an increase in the fluidity of the material (from 6.9 to 27.7 g/10 min) by increasing the LIG content. The Young's modulus and the tensile deformation of the material were practically unaffected when LIG was added. Infrared spectroscopy and differential scanning calorimeter confirmed that there were interactions between LIG and PBS. The DPPH assay was used to evaluate the antioxidant properties of the materials. The results suggested that all the materials were capable of reducing the DPPH concentrations up to 80% in <5 h. Finally, LIG-containing composites showed resistance to adherence of the common nosocomial pathogen, *Staphylococcus aureus*. All tested materials showed ca. 90% less bacterial adherence than PBS.

## Highlights

• Lignin/Poly(butylene succinate) composites were prepared using hot melt extrusion.
• The composites containing up to 15% of lignin.
• The presence of lignin in the composite did not alter significantly their mechanical properties.
• Lignin-containing composites showed antioxidant activity.
• Lignin-containing composites showed resistance to *Staphylococcus aureus* adherence.

## 1 Introduction

The demand for polymer-based materials is increasing due to the growth of the human population and industrial development. The main source for these materials is the petrochemical industry and, therefore, their production has a vast environmental impact [1]. Accordingly, scientists are working on the development of sustainable and green alternatives to conventional polymeric materials [2]. Among other renewable alternatives, materials based on lignin (LIG) have been attracting the attention of researchers [[2], [3], [4], [5], [6], [7], [8], [9], [10]].

LIG is a natural polymer formed by phenyl propane monomers that is present in the cell walls of vascular plants [2,[11], [12], [13]]. This biopolymer provides resistance to various stresses and mechanical support to the cell walls of the plants [2]. Moreover, LIG presents antimicrobial and antioxidant properties [[14], [15], [16], [17], [18]]. LIG is one of the most abundant biopolymers on earth (second after cellulose) [[19], [20], [21]]. Considering all these factors, LIG is a promising candidate to be used as a renewable material. However, the applications of LIG for material production remain unexploited as only 2% of the ca. 70 million tons of LIG produced is re-used for speciality products [2]. The majority of LIG produced is burned as low-grade fuel or just treated as waste [2,22].

Taking into account its high availability and its antioxidant/antimicrobial properties, LIG has great potential to be used in the production of functional medical materials. Up to the present time, the biomedical applications of LIG remain relatively unexplored. Only a few recent works describe the potential use of this biopolymer for healthcare applications. These applications include: additive for tablet manufacturing [23], antimicrobial hydrogels/coatings [24,25], 3D printed antioxidant wound dressing [26] or nanoparticles for drug delivery [27].

The antioxidant and antimicrobial properties of LIG make this biopolymer a good candidate to be included in medical materials. First of all, the uncontrolled production of reactive oxygen species and/or free radicals has been connected with the onset of several diseases such as rheumatoid arthritis, cancer and atherosclerosis [28]. Therefore, the development of antioxidant biomaterials will contribute to reduce the concentration of these species in the body [29]. On the other hand, the antimicrobial properties of LIG can contribute to reduce the risk of bacterial colonization of the surface of the material. Considering that the treatment of medical material-associated infection contributes to the emergence of antibiotic-resistant bacteria [30,31], the development of anti-infective materials will help to address this problem.

In the present research work, the preparation of a combination of LIG and a polymer, poly(butylene succinate) (PBS) by hot melt extrusion for potential biomedical applications is described. PBS is a biodegradable, biocompatible aliphatic polymer [[32], [33], [34]]. This polymer and LIG have been combined before [[35], [36], [37], [38]]. However, LIG/PBS composites have never been explored for potential biomedical applications. In the present work LIG/PBS composites were prepared and characterised. Finally, the antioxidant and antimicrobial properties of the material were evaluated.

## 2 Materials and methods

### 2.1 Materials

The polymer used as the matrix to perform the different test specimens was PBS and it was kindly provided by Natureplast (Ifs, France). LIG sample (BioPiva 100) was a softwood kraft lignin acquired by UPM (Helsinki, Finland). To remove the high moisture content, LIG was placed into the oven at 80 °C for 48 h. The physicochemical properties of this type of LIG can be found in a previously published paper [23]. DPPH (2,2-Diphenyl-1-picrylhydrazyl) was provided by Sigma Aldrich (Dorset, UK). *Staphylococcus aureus* (ATCC 6538; LGC Standards, Middlesex, UK) was maintained on cryopreservative beads in 10% glycerol at −80 °C and cultivated in Mueller Hinton Broth (MHB) at 37 °C when required for the microbiological assessments.

### 2.2 Composite processing and characterization

LIG was incorporated in different percentages (up to 15% w/w) into the PBS matrix. This process was carried out by means of a Brabender plastograph internal mixing machine (Brabender GmbH & Co KG, Brabender Plastograph EC, Germany). The working parameters were established at 150 °C, 80 rpm and 10 min. Once the different obtained materials were cooled, they were milled using a blade mill (IKA, MF 10.2 Impact grinding, France) to produce the pellets, which were dried and stored until further processing. The physical characterization of the obtained materials (pellets) was carried out by means of the melt flow index (MFI) and density tests. MFI was carried out on a CEAST plastometer (Pianezza, Italy) equipped with two thermal resistors that heat a capillary. This test was carried out at 175 °C with a weight of 2.16 kg, measuring the amount of melted and discharged material in 10 min, according to the standard ISO 1133. Density was measured by means of a pycnometer following the ISO standard ISO 1183-1.

Then, the PBS and LIG compounds were injection-moulded in a 220 M 350-90 U injection machine (Aurburg, Germany). The injection process was carried out with a first pressure of 120 kg/cm^2^ and a second pressure of 37.5 kg/cm^2^. The injection temperatures in the different zones of the injection machine were 150, 160, 170 and 175 °C, corresponding the last temperature to the injection nozzle. Ten standard test specimens from every LIG-PBS blend were used for each mechanical test. The standard specimens used for tensile tests were 160 × 13.3 × 3.2 mm according to ASTM D638 and 127 × 12.7 × 3.2 mm for flexural and impact tests according to ASTM D3641. Standard specimens were kept in a climatic chamber (Dycometal, Spain) at 50% relative humidity and 23 °C for at least 48 h before testing. The tensile and flexural properties of the samples were tested using an Instron TM 1122 universal testing machine (Norwood, United States of America) equipped with a 5 kN load cell and an MFA2 extensometer for higher deformation accuracy. The test speed was 2 mm/min as established by ASTM D790. The tests of the impact properties were carried out with the Charpy pendulum (Madrid, Spain) with and without notch and the Izod pendulum according to the ISO 178 standard.

To proceed with the materials characterization, the thermal properties of the different test specimens, glass transition temperature (T_*g*_) and the melting temperature (T_m_) were assessed using DSC Q100 differential scanning calorimeter (TA instruments, Bellingham, WA, USA). Scans were run from −70 to 30 °C and from 30 to 350 °C at 10 °C/min under a nitrogen flow rate of 50 mL/min, for the T_*g*_ and T_m_, respectively.

The Fourier Transform Infrared (FTIR) spectra of the different test specimens were recorded using a Spectrum Two™ instrument (Perkin Elmer, Waltham, MA, USA) by the attenuated total reflectance (ATR) technique. The spectra were recorded from 4000 cm^−1^ to 400 cm^−1^ with a resolution of 4 cm^−1^ and a total of 32 scans were collected.

The contact angle of water with the surface of the different blends was evaluated using an Attension Theta equipment (Attension Theta, Biolin Scientific, Gothenburg, Sweden). For this purpose 4 μL of water were dropped into the material surface and the contact angle was measured after 1.94 s. OneAttension software analysed results to give an indication of the wettability of the surface. Measurements were performed in triplicate. The morphology of the different obtained test specimens was evaluated by using scanning electronic microscopy (SEM) (Hitachi TM3030; Tokyo, Japan) in EDX mode. Images were recorded using a magnification of ×300.

Finally, a stability study was performed. For this study, replicates of the test specimens (0.13–0.18 g) containing different LIG proportions were incubated at 37 °C in screw-capped vials containing 3 mL of phosphate-buffered saline (pH = 7.4) over a period of 45 days. After different interval times, the samples were separated from the degradation medium and the excess of it was removed with a tissue paper and subsequently they were dried at 80 °C in the oven to constant weight. The degradation medium or phosphate-buffered saline was replaced after each time interval and the mass loss was calculated using Eq. (1).

(1) $$\%\text{weight loss}=100\;\left(w_{0}-w_{t}\right)/w_{0}$$

where w_0_ is the initial weight of the composite and w_t_ is the weight of the sample at a defined time.

### 2.3 Antioxidant properties

DPPH (2,2-diphenyl-1-picrylhydrozyl) radical was employed to measure the antioxidant activity of the different test specimens [39]. Briefly, 3 mL of a DPPH solution dissolved in methanol (23.6 mg/L) was added to the test specimens (a square of 1 cm × 1 cm × 0.32 cm) placed in a vial. A control sample of 23.6 mg/L of DPPH in methanol was also measured. For test specimens containing 5, 10 and 15% LIG a more concentrated DPPH solution (47.2 mg/L) was also used to measure their antioxidant activity. The samples were then incubated in the dark for 300 min at room temperature. At predetermined time intervals (each 60 min), 300 μL sample was collected and the vials were immediately replenished with an equivalent volume of methanol. The absorbance of the different solutions was measured at 517 nm in triplicate using a UV–vis plate reader (PowerWave XS Microplate Spectrophotometer, Bio-Tek, Winooski, USA). The radical scavenging activity was calculated as Eq. (2).

(2) $$\mathrm{RSC}\;\left(\%\right)=100\;\left(A_{0}-A_{1}/A_{0}\right)$$

where A_0_ = absorbance of the control sample and A_1_ = absorbance in the presence of the sample at any time. Decreased absorbance of the reaction indicates a stronger DPPH radical scavenging activity.

### 2.4 Antibacterial properties

The in vitro microbiological analysis was performed according to the previous published works [[40], [41], [42]]. In brief, a bacterial suspension of *S*. *aureus* (1 × 10^8^ cfu mL^−1^) in phosphate-buffered saline and supplemented with 0.5% of tryptone soya broth (TSB) (pH 7), was diluted (1:100) with phosphate-buffered saline containing 0.5% TSB. Replicate samples of the test specimens (a square of 1 cm × 1 cm × 0.32 cm) were placed in individual wells of a 24-well plate and then aliquots of 1 mL of the diluted bacterial suspension with a density of 1 × 10^6^ cfu mL^−1^ were added, ensuring the test specimens were completely covered. The plate was continuously shaken at 100 rpm in an orbital incubator at 37 °C for 24 h. Then, the samples were removed from the 24-well plate containing the bacterial suspension and the nonadherent bacteria were removed by serial washing, first in phosphate-buffered saline (1 × 10 mL), and secondly, in quarter-strength Ringers Solution (QSRS) (3 × 10 mL) [43]. After the wash step, test specimens were transferred into fresh QSRS (5 mL), sonicated (15 min) and vortexed (30 s) to remove adherent bacteria. The sonication technique has previously been demonstrated not to affect bacterial viability or morphology [44]. A viable count of the QSRS was performed by the Miles and Misra serial dilution technique [45] followed by plating onto Mueller-Hinton agar to enumerate the previously adhered bacteria per sample.

## 3 Results and discussion

### 3.1 Surface and physio-mechanical properties of LIG/PBS composites

**Fig. 1:**
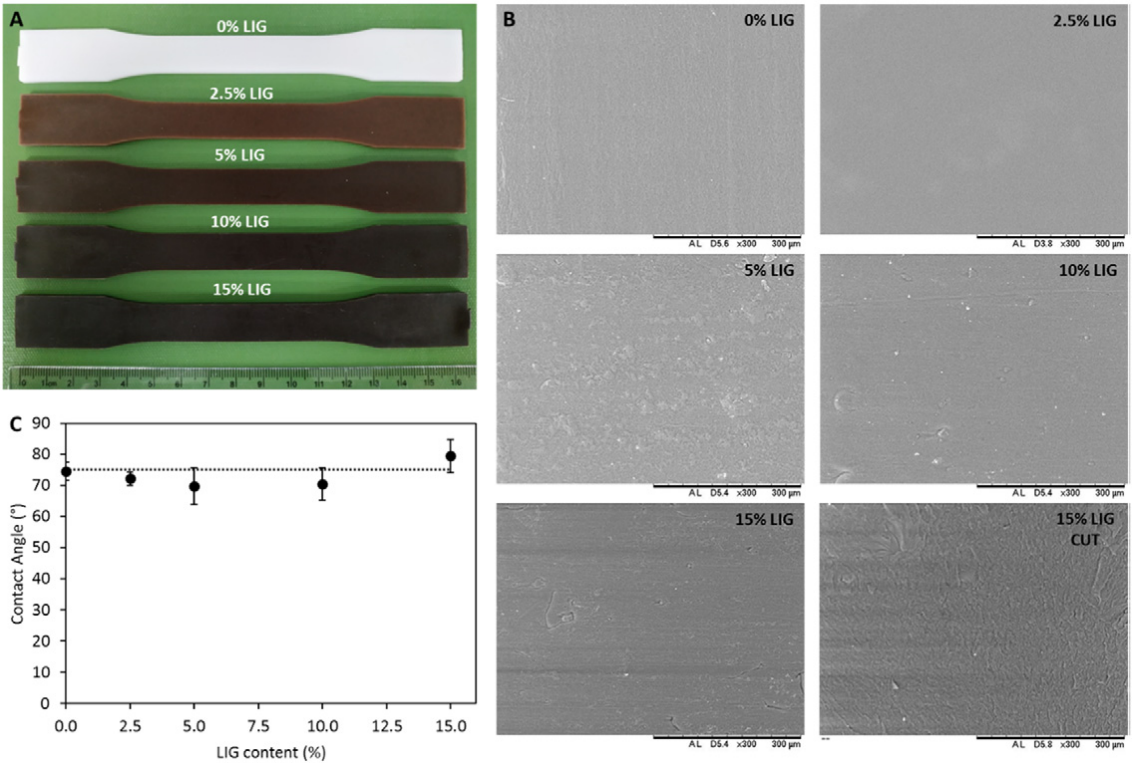
Images of PBS test specimens prepared using different LIG concentrations (A). SEM images of the surface of the PBS/LIG materials and a transversal cut of the sample with 15% LIG (B). Contact angle as a function of the LIG content for all the materials (C).

Fig. 1A shows the test specimens prepared using PBS and LIG. The materials showed a homogeneous distribution of LIG through the material. There were no obvious sections where LIG accumulated or any visible LIG particles. Moreover, it can be observed that higher LIG concentrations yielded a darker material with a stronger brown colour. This is due to the native brown/black colour of LIG. This suggests a good mixing between PBS and LIG. However, in order to evaluate the miscibility of both materials, chemical analysis was performed (see Section 3.2).

In order to evaluate the surface morphology of the materials SEM was used (Fig. 1B). It can be seen that the inclusion of LIG yielded slightly more rugged surfaces. This is not obvious for materials containing 2.5% (w/w) of LIG. However, when the biopolymer concentration was increased the surface showed a more rugged structure. Moreover, Fig. 1B shows a transversal cut of the composite containing 15% (w/w) of LIG. No LIG aggregates were observed within the materials confirming that hot-melt extrusion yielded homogeneous mixture for PBS and LIG.

Contact angle measurements were used to gain more information about the surface properties of the materials. The contact angle of water with the surface of the materials is shown in Fig. 1C. It can be seen that there is not a clear trend. All the evaluated materials showed similar contact angle values. Moreover, statistical analysis suggested that the differences between all these measurements were not significant (p > 0.05). Accordingly, it can be considered that the wettability of the materials was not affected by the incorporation of LIG.

The obtained contact angles for pure PBS are similar to the ones previously reported for poly(lactic acid), a similar biodegradable polymer [46]. Moreover, Ye et al. described that the combination of LIG and poly(lactic acid) yielded more wettable materials (lower contact angle) [46]. However, in the case of PBS this effect was not observed.

**Fig. 2:**
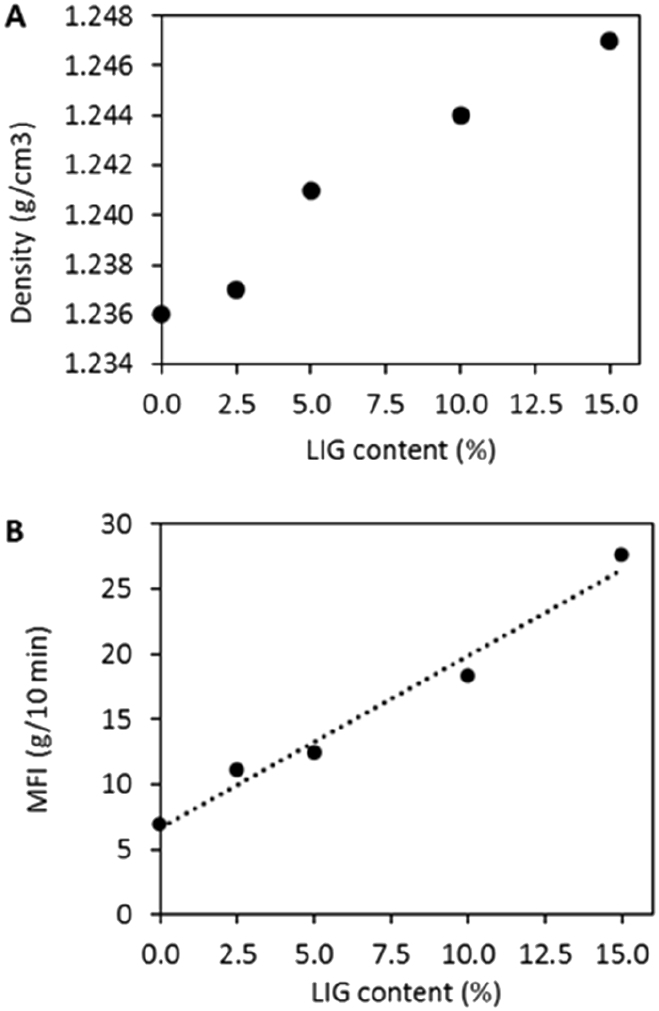
Effects of lignin content on density (A) and MFI (B).

By assessing the melt flow index and the density of the different produced materials it is possible to evaluate to a certain extent the processability of these materials. The incorporation of powdered LIG in the production of PBS matrix materials caused virtually no change in the density of the material. The density increases in the materials with the higher LIG content (15% w/w) was only 0.011 g/cm^3^ (<1%) with respect to the PBS matrix (from 1.236 to 1.247 g/cm^3^) (Fig. 2A). This low effect on the density of the material would allow its incorporation into materials without increasing the weight of the final piece, as has been previously reported using organo-montmorillonite [47].

On the other hand, the melt flow index test (Fig. 2B) showed an increase in the fluidity of the material by increasing the LIG content. These results were surprising since the incorporation of mineral charges such as talc or kaolin, with a low aspect ratio or fibers for reinforcement, considerably decreases the fluidity of the material [48,49]. In this sense, the most plausible explanation is the plasticization of the LIG, giving rise to thermoplastic LIG which produces an increase in the fluidity of the material. During the composite extrusion process, LIG at elevated temperatures can plasticize giving rise to a thermoplastic material with high flowability, as has been reported [50]. Therefore, this increase in the fluidity index of the material could allow in future applications the addition of reinforcements such as natural fibers without loss of processability.

The tensile properties (tensile strength *σ*_*t*_^*C*^, Young's modulus *E*_*t*_^*C*^, and tensile deformation *ε*_*t*_^*C*^) of the different materials produced represented in the Table 1 showed a similar behavior to that which has been reported previously by other authors when they added LIG in a polypropylene matrix [51]. The tensile strength decreased as a greater amount of LIG was incorporated, due to the low aspect ratio that this aromatic polymer presents, acting as a filler. However, the stiffness of the material represented by Young's modulus and the tensile deformation were practically unaffected, maintaining values close to those obtained for the PBS matrix (0.79 GPa and 17.85%, respectively). These results together with the small loss of flexural strength (*σ*_*f*_^*C*^) (<10% for the maximum LIG content) allow us to expect a good behavior of this material when used for biomedical applications such as catheters or anti-infective materials, according to the results reported by Thakur et al. 2014 or Taurino et al. 2018 [21,52]. In this sense, the results obtained showed that the properties of the flexural modulus (*E*_*f*_^*C*^) and the flexural elongation (*ε*_*f*_^*C*^) are maintained contrary to what usually happens when adding mineral fillers [48].

**Table 1:** Mechanical properties of composites with different lignin content.

LIG content (%)	Tensile properties		
	*E*_*t*_^*C*^(GPa)	*σ*_*t*_^*C*^(MPa)	*ε*_*t*_^*C*^(%)
0.0	0.79 ± 0.02	39.37 ± 0.28	17.85 ± 0.02
2.5	0.77 ± 0.01	37.25 ± 0.16	18.51 ± 0.16
5.0	0.77 ± 0.03	35.53 ± 0.49	17.90 ± 0.17
10.0	0.77 ± 0.01	32.82 ± 0.14	17.16 ± 0.08
15.0	0.80 ± 0.02	30.69 ± 0.22	15.23 ± 0.35


LIG content (%)	Flexural properties		
	*E*_*f*_^*C*^(GPa)	*σ*_*f*_^*C*^(MPa)	*ε*_*f*_^*C*^(%)
0.0	0.60 ± 0.04	35.14 ± 0.40	12.53 ± 0.23
2.5	0.60 ± 0.05	33.66 ± 0.09	12.83 ± 0.18
5.0	0.59 ± 0.05	32.81 ± 0.43	12.68 ± 0.15
10.0	0.58 ± 0.03	31.73 ± 0.40	12.67 ± 0.13
15.0	0.60 ± 0.05	31.52 ± 0.19	12.83 ± 0.18


LIG content (%)	Impact properties		
	Charpy		Izod (J/m)
	Without notch (KJ/m^2^)	With notch (KJ/m^2^)	
0.0	Not break	8.96 ± 0.26	87.46 ± 4.22
2.5	Not break	6.46 ± 0.34	61.92 ± 5.43
5.0	Not break	6.19 ± 0.15	54.47 ± 1.49
10.0	Not break	5.04 ± 0.43	41.98 ± 2.92
15.0	Not break	2.48 ± 0.23	28.80 ± 3.01

Finally, the impact properties were evaluated for Charpy pendulums with and without notch and Izod. The results showed a significant decrease in the impact resistance of these materials when the LIG content was increased. This phenomenon is common for composite materials where particles or natural fibers are incorporated [53]. However, the samples tested without notch continue to show good resistance to impact since in none of the cases they were broken. In short, the addition of LIG provided an environmentally friendly material without loss of the desired characteristics of the virgin PBS. Therefore, it is clear that LIG has great potential as a filler and reinforcement for composites requiring similar properties to PBS.

### 3.2 Chemical characterization of LIG/PBS composites

**Fig. 3:**
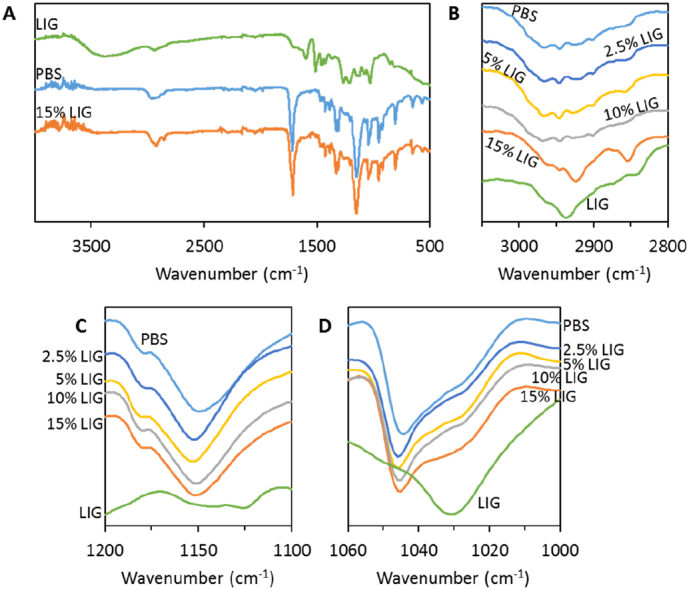
FTIR spectra of LIG, pure PBS and PBS containing 15% (w/w) LIG (A). Expanded areas of the FTIR spectra for PBS/LIG materials (B-D).

Fig. 3 shows the FTIR spectra for the PBS/LIG materials. The full FTIR spectra of LIG, PBS and PBS containing 15% (w/w) of LIG can be seen in Fig. 3A. The infrared spectra of the composites containing LIG show the same main peaks present in the PBS spectrum (Fig. 3A). However, LIG containing composites present some differences in their FTIR spectra when compared with PBS spectra. Fig. 3B shows a magnified section of the FTIR spectra between 2800 and 3050 cm^−1^. Composites containing up to 10% (w/w) LIG showed a broad peak that is present in PBS spectra. However, when LIG concentration reached 15% (w/w) two sharper peaks were found at around 2925 and 2840 cm^−1^. These peaks were not observed in the other composites due mainly to the low LIG content. Similar peaks can be found in pure LIG (Fig. 3B). Nevertheless, these peaks appeared at slightly different wavenumbers in the spectrum of pure LIG. These bands can be attributed to the CH stretching of methylene groups of side chains and aromatic methoxyl groups [35,54,55]. Moreover, the shifts observed for these two peaks in the composite suggest that a certain degree of interaction between PBS and LIG occurs when they are combined using hot melt extrusion. The PBS carbonyl peak (ca. 1700 cm^−1^) did not show any shift when the polymer was combined with LIG (data not shown). Nevertheless, the C-O-C stretching of the ester bonds in PBS (ca. 1150 cm^−1^) showed a chemical shift to higher wavenumbers when LIG was incorporated into the material (Fig. 3C). Again, this confirms that there are some non-covalent interactions between PBS and LIG. Similar behavior has been reported before for PBS and polydioxanone blends [56]. This is consistent with the findings obtained by Saffian et al. that reported the presence of hydrogen bonds between PBS and LIG [57]. Finally, LIG showed a peak ca. 1030 cm^−1^ that can be attributed to aromatic C=C stretching (Fig. 3D) [35]. The presence of this peak in the composites can be observed in Fig. 3D. The intensity of this peak increased with the LIG concentration in the material. However, no shift was observed for this particular peak.

**Fig. 4:**
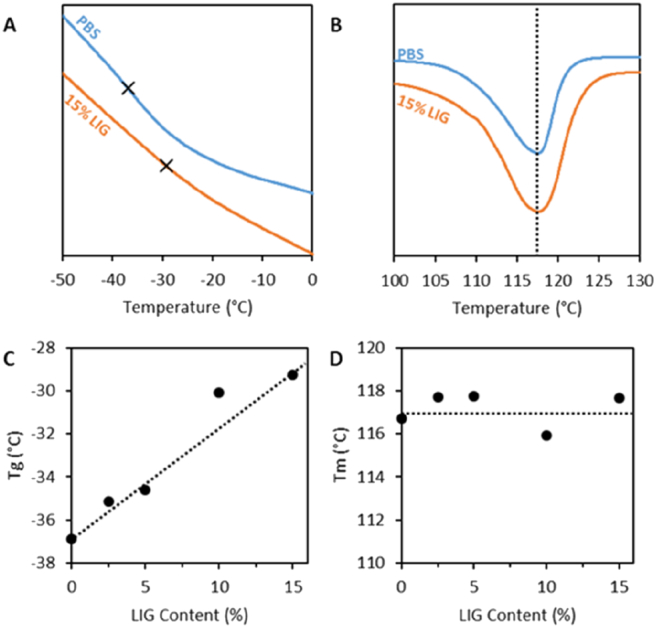
PBS and PBS containing 15% (w/w) LIG DSC thermograms showing the T_*g*_ (A) and T_m_ (B) regions. T_*g*_ (C) and T_m_ (D) as a function of LIG content.

Fig. 4A and B shows the DSC thermograms of PBS and PBS containing 15% (w/w) of LIG. Only the thermograms of the composite containing 15% (w/w) of LIG were displayed for clarity purposes. It can be seen that the T_*g*_ shifts to higher values when LIG was incorporated into the material (Fig. 4A) while the T_m_ did not show any changes (Fig. 4B). The T_*g*_ changes can be correlated with LIG content as shown in Fig. 4C. On the other hand, T_m_ was not affected by the LIG content within the material (Fig. 4D). These results confirm what FTIR results suggested, there are interactions between LIG and PBS.

Previous studies suggested that LIG acts as a nucleating agent for PBS and poly(3-hydroxybutyrate) [35,58]. Sahoo et al. reported changes in PBS T_*g*_ when combined with LIG using hot melt extrusion [35]. However, in this work increasing the LIG content up to 30% increased the T_*g*_ by 5 °C. In the present work, with only 15% of LIG the T_*g*_ of the material was increased in ca 7 °C. Moreover, Sahoo et al. reported that when the concentration of LIG in the materials increased, the T_m_ decreased slightly [35]. This effect was not observed in the present work. However, in order to observe this effect Sahoo et al. increased the LIG content up to 65% [35]. Interestingly, Lin et al. reported that the combination of PBS with calcium lignosulfonate (LIG derivative) has the opposite effect as it contributes to reduce the T_*g*_ of the polymers [38]. Interestingly, the combination of lignosulfonate and PBS alters the crystallinity of PBS [38]. It was described that composites containing up to 10% of lignosulfonates showed slightly higher T_g_ values. Lin et al. suggested that the rigid LIG-based filler inhibited the movement of the PBS segments improving the crystallinity of the composite [38]. When up to 10% (w/w) of the lignosulfonates were combined with PBS there were only slight changes on the T_m._ However, no clear trend was observed. This behavior is similar to the one reported in the present work.

Finally, the degradation kinetic experiments showed that neither PBS nor PBS/LIG composites showed any significant weight change over 45 days at 37 °C in phosphate-buffered saline (p > 0.05) (data not shown). With the available results we can conclude that LIG did not affect the degradability of the material under those conditions.

### 3.3 Antioxidant properties of the LIG/PBS composites

**Fig. 5:**
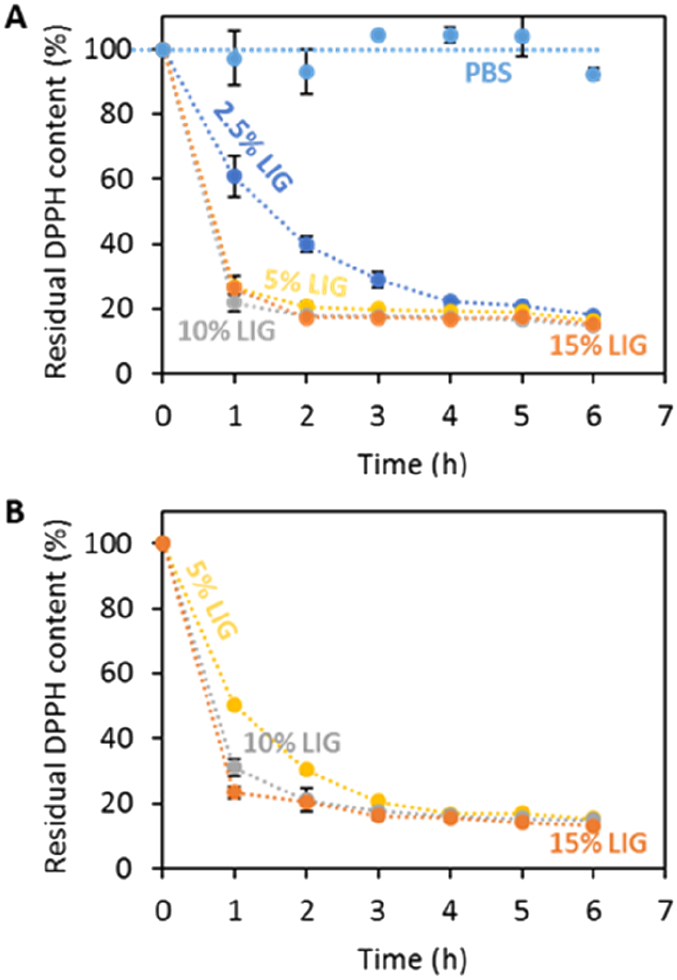
Residual DPPH content as a function of time for PBS/LIG based materials using an initial DPPH concentration of 23.6 mg/L (A). Residual DPPH content as a function of time for PBS/LIG based materials containing 5–15% (w/w) LIG using an initial DPPH concentration of 47.2 mg/L (B).

In order to check the antioxidant properties of the PBS/LIG composites, DPPH assay was carried out (Fig. 5A). This assay showed that PBS did not show any antioxidant activity. When immersing a piece of PBS into a DPPH solution the concentration of this organic compound remained unchanged during the testing time (6 h). However, when LIG was incorporated into the material the antioxidant activity of the material increased. Fig. 5A shows how composites containing 2.5% (w/w) of LIG were capable of reducing DPPH concentration over time reaching its maximum effect after 5 h. Moreover, higher concentrations of LIG in the material showed the same antioxidant activity as they were able to noticeably reduce the DPPH concentration up to ca. 80% after 2 h (Fig. 5A).

Fig. 5B shows the DPPH assay carried out with a higher DPPH concentration to evaluate if there is any difference between the antioxidant capabilities of the composites containing between 5 and 15% (w/w) of LIG. As expected, higher LIG concentration yielded composites with a higher antioxidant activity during the first two hours of the study. Again, after 3 h all the composites reached its maximum antioxidant activity.

Similarly, other authors have studied the antioxidant properties of LIG-based composites. Domínguez-Robles et al. combined poly(lactic acid) (PLA) with LIG for wound care 3D printing applications [26]. DPPH concentration reductions of up to 80% after 5 h were reported in this work. However, the composites reported by Domínguez-Robles et al. contained a maximum of 3% of LIG. These results are similar to the ones reported in the present paper for composites containing 2.5% LIG. On the other hand, Kai et al. reported the antioxidant properties of poly(lactic acid) LIG nanofibers [59]. However, Kai et al. obtained DPPH concentration reductions up to ca. 60% after 72 h using the same initial concentration of DPPH used in this study (23.6 mg/L) for materials containing up to 50% (w/w) of LIG [59]. In the present study, with a loading of 2.5% (w/w) of LIG superior antioxidant properties were obtained.

Antioxidant materials have potential for biomedical applications as they contribute to reduce the concentration of reactive oxygen species and free radicals [8,60]. Moreover, the uncontrolled production of these compounds is connected with the development of diseases such as cancer, atherosclerosis or rheumatoid arthritis [28]. In addition to the development of these conditions, it has been reported that high concentrations of reactive oxygen species precludes wound healing [61,62]. Accordingly, antioxidant materials can contribute to accelerate wound healing.

### 3.4 Antibacterial properties of the LIG/PBS composites

In addition to the antioxidant properties, LIG has been reported to have antibacterial properties [4,17,24]. Accordingly, the LIG-based composites are expected to retain this property. Bacterial adherence to the surface of the composites was studied with the Gram-positive *S*. *aureus* as a model pathogen. This pathogen was selected since LIG has previously been reported to have greater activity against Gram-positive microorganisms than Gram-negative bacteria [17]. Moreover, *S*. *aureus* is a common causative agent of medical device and bloodstream infections [63].

**Fig. 6:**
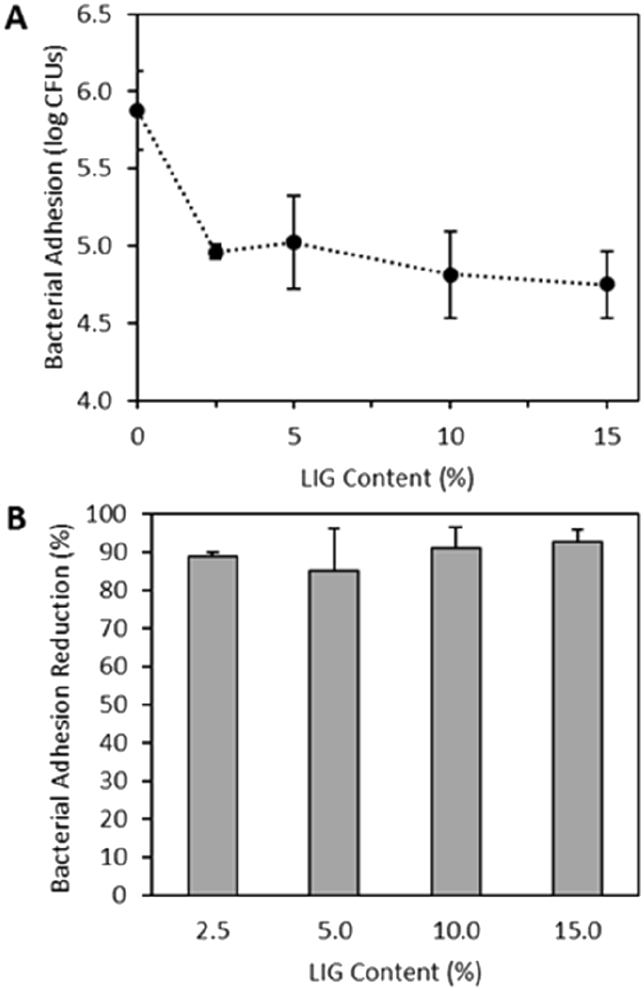
*S*. *aureus* adhesion to PBS/LIG materials as a function of LIG content (A). *S*. *aureus* adhesion reduction relative to PBS control for the PBS/LIG materials (B).

It can be seen that composites containing LIG showed significantly greater resistance to adherence of *S*. *aureus* than PBS (Fig. 6A) (p < 0.05). Interestingly, the results suggested that the resistance to bacterial adherence was independent of the LIG content as all the composites showed a similar degree of adherent bacteria (p > 0.05). When compared with PBS the materials showed a reduction in adherent bacterial cells of ca. 90% (Fig. 6B). These results are extremely promising considering that anti-infective materials have potential to be used for biomedical applications.

A previous work that studied the combination of PLA with LIG described that the resulting composites did not show any antimicrobial properties [26]. LIG content within the composites in this study was up to 3% (w/w). Considering the results obtained for PBS/LIG composites containing 2.5% (w/w) of LIG this suggest that the use of PBS instead of PLA provides added value to the resulting materials. These results are interesting as PLA is a biodegradable polymer [64] similar to PBS. However, the mixing procedure used to combine PLA and LIG was different and can be the reason that explains the lack of antibacterial properties for PLA/LIG composites.

Nosocomial infections are caused by bacterial attachment to surfaces of implanted or indwelling medical devices that results in biofilm formation [65]. These infections are hard to treat as they are resistant to antibacterial treatment [66]. Accordingly, they prolong hospital stays while increasing patient morbidity/mortality and healthcare costs [66]. Therefore, extensive efforts have been made during recent years to develop antimicrobial materials [40,52,[67], [68], [69]]. Despite its antibacterial activity there is only one published work that describes the use of LIG for potential biomedical applications [24]. Accordingly, the findings presented here are promising for future applications of LIG-based composites in biomedical applications.

## 4 Conclusions

The present work described the preparation and characterization of LIG and PBS composites. Moreover, the antioxidant and antimicrobial properties of these materials were evaluated. The obtained results showed that these two polymers can be combined using hot-melt extrusion. The resulting materials contained up to 15% (w/w) of LIG. The combination of LIG with PBS yielded composites with similar stiffness to the one measured for PBS. Additionally, the composites showed a small loss of flexural strength when LIG was added. FTIR and DSC confirmed that there were interactions between PBS and LIG. Finally, the antimicrobial and antioxidant properties of the composites were evaluated. The results showed that LIG containing materials showed both antimicrobial and antioxidant properties. Furthermore, only a small amount of LIG (2.5%) was required to achieve both activities.

It is important to note that the process used to prepare PBS/LIG composites is environmentally friendly as no organic solvent was required. Moreover, PBS is a biodegradable polymer and LIG is a biopolymer from renewable sources. Accordingly, the resulting composites can be considered green materials.

The composites presented in the present work have potential for biomedical applications due to their antibacterial and antioxidant properties. The uses of LIG for pharmaceutical and biomedical applications have been barely explored. In order to achieve its full potential, more research needs to be conducted and this biopolymer will need approval by regulatory bodies, such as the US Food and Drug Administration (FDA), for pharmaceutical/biomedical applications. The results presented here and in other work presented by other authors are promising first steps in achieving this.

## CRediT authorship contribution statement

**Juan Domínguez-Robles:** Conceptualization, Methodology, Investigation, Formal analysis, Data curation, Writing - review & editing, Supervision, Funding acquisition. **Eneko Larrañeta:** Conceptualization, Methodology, Data curation, Writing - review & editing, Funding acquisition. **Mun Leon Fong:** Investigation, Formal analysis. **Niamh K. Martin:** Investigation, Formal analysis. **Nicola J. Irwin:** Methodology, Funding acquisition. **Pere Mutjé:** Supervision. **Quim Tarrés:** Conceptualization, Writing - review & editing. **Marc Delgado-Aguilar:** Methodology, Investigation, Formal analysis.
